# Racial/Ethnic Differences in Influenza and Pneumococcal Vaccination Rates Among Older Adults in New York City and Los Angeles and Orange Counties

**DOI:** 10.5888/pcd15.180101

**Published:** 2018-12-13

**Authors:** Stephanie C. Tse, Laura C. Wyatt, Chau Trinh-Shevrin, Simona C. Kwon

**Affiliations:** 1 NYU School of Medicine, Department of Population Health, New York, New York

## Abstract

**Introduction:**

Disparities in vaccination rates exist among racial/ethnic minority adults. This study examined factors associated with influenza (flu) and pneumococcal vaccination rates among non-Hispanic black, Hispanic, and Asian American adults aged 50 or older living in New York City or Los Angeles and Orange counties in California.

**Methods:**

We used data collected by the REACH US Risk Factor Survey 2009–2012 in New York City and California. We analyzed data on 14,139 adults aged 50 or older who were categorized as non-Hispanic black (New York City [n = 1,715], California [n = 530]), Hispanic (New York City [n = 2,667], California [n = 1,099]), Chinese American (New York City [n = 1,656]), Korean American (New York City [n = 310]), Filipino American (California [n = 1,515]), or Vietnamese American (California [n = 3,435]). Bivariate analyses examined difference across race/ethnicity and location, and multivariable logistic regression models, adjusting for sociodemographic and health variables, examined flu and pneumococcal vaccination rates.

**Results:**

Among adults aged 50 or older, the flu vaccination rate was lower among non-Hispanic black respondents (New York City, 53.3%; California, 40.5%) than among Hispanic (New York City, 61.0%; California, 49.4%), Chinese (New York City, 67.6%), Korean (New York City, 60.5%), Filipino (California, 66.2%), and Vietnamese (California, 68.0%) respondents. Among adults aged 65 or older, pneumococcal vaccination rates were lowest among Chinese and Korean respondents in New York City (51.7% and 49.1%, respectively), compared with non-Hispanic black (New York City, 62.0%, California, 65.6%), Hispanic (New York City, 60.0%; California 62.7%), Filipino (California, 63.4%), and Vietnamese (California, 63.8%) respondents. Older age, having had a checkup in the past year, and diabetes diagnosis were significantly associated with flu and pneumococcal vaccination in both locations. Additional variables were significant for some vaccinations and locations.

**Conclusion:**

When compared with Asian American respondents, non-Hispanic black respondents were least likely to receive the flu vaccine in New York City and California. We found no racial/ethnic differences in pneumococcal vaccination rates. Our findings highlight the need for targeted efforts to increase vaccination rates among racial/ethnic minority older adults.

## Introduction

Influenza (flu) and pneumococcal vaccinations offer important protection against flu complications and pneumococcal diseases, which can become life threatening in vulnerable populations such as children, persons with chronic conditions, and older adults. The Centers for Disease Control and Prevention (CDC) recommends that everyone aged 6 months or older receive a flu vaccination every season (1). CDC also recommends that everyone aged 65 or older receive both conjugate and polysaccharide pneumococcal vaccines (2). Healthy People 2020 goals include increasing the percentage of noninstitutionalized adults aged 65 or older receiving the seasonal flu vaccine and pneumococcal vaccine to 90.0% (3).

Overall vaccination rates among adults aged 65 or older were below the goal of 90%, according the 2014 National Health Interview Survey, and national and state studies demonstrate differences in vaccination rates by race/ethnicity. For instance, according to various surveys in recent years, non-Hispanic white adults had the highest rates of annual flu vaccination nationally (range, 68.0%–75.1%), compared with non-Hispanic black (range, 53.0%–64.3%), Hispanic (range, 57.5%–64.1%), and Asian American (range 65.2%–83.5%) adults (4–7). Similarly, rates of ever receiving a pneumococcal vaccination were higher among non-Hispanic white adults (range, 61.1%–71.1%) than among non-Hispanic black (range, 38.9%–57.7%), Hispanic (range, 32.0%–51.9%), and Asian American adults (range, 41.3%–49.0%) (4,5,7–9). State data demonstrate additional disparities. Flu vaccination rates in California among adults aged 65 or older were highest among Asian American adults (range, 64.9%–80.1%), followed by Hispanic (range, 52.6%–67.5%), non-Hispanic white (59.1%), and non-Hispanic black (range, 46.2%–59.5%) adults; whereas rates of ever receiving a pneumococcal vaccination among adults aged 65 or older were highest among non-Hispanic white adults (range, 67.0%–77.8%), followed by Asian American (range, 56.0%–73.0%), non-Hispanic black (range, 61.8%–68.3%), Hispanic (range, 48.2%–58.3%), and Vietnamese American adults (41.0%) (10–12). Among adults aged 50 or older, Vietnamese Americans had higher annual flu vaccination rates (60.6%) than non-Hispanic white (51.7%) and Asian American adults (45.3%) (11). Flu vaccination rates in New York State among adults aged 65 or older were highest among Asian Americans (range, 62.1%–83.4%), followed by non-Hispanic white (60.5%), Hispanic (range, 58.3%–67.6%), and non-Hispanic black adults (range, 52.1%–63.2%), whereas rates of ever receiving a pneumococcal vaccination among adults aged 65 or older were highest among non-Hispanic white (72.9%), followed by Hispanic (59.9%–66.1%), Asian American (52.4%–57.0%), and non-Hispanic black (52.7%–53.2%) adults (10–12).

These studies consistently showed that racial/ethnic minority groups have lower vaccination rates than non-Hispanic white adults. In the aggregate, Asian Americans are often found to have vaccination rates similar to rates among non-Hispanic white adults. This aligns with the “model minority” stereotype that posits Asian Americans are doing well socioeconomically and are adhering to healthy behaviors compared with other racial/ethnic groups (7,9,13). Aggregated data on Asian American health, however, mask large and significant differences that exist across Asian subgroups (14,15). Asian Americans comprise more than 50 ethnicities, each of which has unique health behaviors, cultural values, and varying degrees of access to health care resources (15–17). The limited data available on vaccination rates among older Asian Americans indicate that pneumococcal vaccination differs by subgroup: Japanese Americans (59.8%), Asian Indian Americans (26.2%), Korean Americans (24.7%), and Filipino Americans (22.9%) (9). Although flu vaccination rates have not been published for Asian American subgroups aged 65 or over, variation exists among adult Asian American subgroups overall. Annual flu vaccination rates among Asian American adults aged 18 or older are the following: Japanese Americans (range, 30.7%–44.1%), Chinese Americans (range, 26.5%–36.6%), Filipino Americans (range, 26.1%–35.9%), Korean Americans (range, 23.4%–48.9%), and Vietnamese Americans (range, 28.7%–46.7%).

To identify factors associated with persistent disparities in vaccination rates among racial/ethnic minority adults aged 50 or older, we compared flu and pneumococcal vaccination rates among older non-Hispanic black, Hispanic, and Asian American populations living in New York City and 2 counties in California, Los Angeles County and Orange County.

## Methods

Racial and Ethnic Approaches to Community Health (REACH) is a national program administered by CDC, beginning in 2007, to mobilize local communities to implement community-based strategies for the elimination of health disparities in racial/ethnic minority populations. The REACH US Risk Factor Survey was conducted annually from 2009 to 2012 to evaluate program activities in 28 REACH communities (12). New York City and Los Angeles and Orange counties in California had large numbers of Asian American, non-Hispanic black, and Hispanic adults; therefore, we studied these locations. The NYU School of Medicine institutional review board policy indicated this research did not involve human participants and that institutional review board review was not required. 

The survey used an address-based sampling design with geographical information systems technology to target US Census tracts with large numbers of Asian American, Hispanic, and non- Hispanic black adults. Questions were derived from the Behavioral Risk Factor Surveillance System (18). Data on 14,139 adults aged 50 or older were categorized as non-Hispanic black (New York City = 1,715, Los Angeles and Orange counties = 530), Hispanic (New York City = 2,667, Los Angeles and Orange counties = 1,099), Chinese American (New York City = 1,656), Korean American (New York City = 310), Filipino American (Los Angeles and Orange counties = 1,515), or Vietnamese American (Los Angeles and Orange counties = 3,435). Details on methods are available elsewhere (12,19,20).

We selected adults aged 50 or older to examine rates of flu vaccination (to increase our sample size and power) and adults aged 65 or older to examine rates of pneumococcal vaccination. We excluded Asian American subgroups with fewer than 100 respondents among adults aged 65 or older.

Outcomes of interest included a flu vaccination in the past 12 months and a lifetime pneumonia vaccination. We used responses to the following 2 questions: “During the past 12 months, have you had a flu shot?” and “Have you ever had a pneumonia shot?”

Independent variables were age group (50–64 or ≥65); sex (male or female); nativity (US born or non-US born), education (<high school diploma, high school diploma/some college, or college graduate); self-reported health (good, fair/poor, or excellent/very good); health insurance (yes or no); most recent checkup (within past year or more than a year/never/do not know); smoking status (current, former, or never); ever received a diagnosis of stroke, angina, or coronary heart disease (yes, no/do not know); needed a physician but was too costly (yes or no); and ever received a diagnosis of diabetes (yes or no). We chose these variables on the basis of literature that identified associations between sociodemographic and health-related factors and vaccination outcomes (4,5,11,21).

### Data analysis

We performed descriptive analyses for all independent variables stratified by location and racial/ethnic minority group. We performed bivariate analyses; we ran χ^2 ^tests (categorical variables) and independent samples *t* tests (continuous variables) to evaluate significant differences of sociodemographic and health-related variables affecting vaccination outcomes among non-Hispanic black respondents, Hispanic respondents, and Asian American subgroups, and to inform variables to be used in the models. Finally, we analyzed racial/ethnic and geographic differences in vaccination receipt by using multivariable logistic regression; we tabulated odds ratios (ORs) and 95% confidence intervals (CIs). Model 1 accounted for sociodemographic factors, and Model 2 (the full model) accounted for sociodemographic and health-related factors. We performed all data analyses by using SAS-callable SUDAAN version 11.0.2 (RTI International), and we used an α level of <.05. Because our study was secondary data analysis of cross-sectional data, we did not conduct a power analysis to determine sample size.

## Results

The rate of receiving a flu vaccination among non-Hispanic black respondents was 53.3% in New York City and 40.5% in Los Angeles and Orange counties; among Hispanic respondents, 61.0% in New York City and 49.4% in Los Angeles and Orange counties; among Chinese American respondents in New York City, 67.6%; among Korean American respondents in New York City, 60.5%; among Filipino American respondents in Los Angeles and Orange counties, 66.2%; and among Vietnamese American respondents in Los Angeles and Orange counties, 68.0% (Table 1). The rate of receiving a pneumococcal vaccination among non-Hispanic black respondents was 62.0% in New York City and 65.6% in Los Angeles and Orange counties; among Hispanic respondents, 60.0% in New York City and 62.7% in Los Angeles and Orange counties; among Chinese American respondents in New York City, 51.7%; among Korean American respondents in New York City, 49.1%; among Filipino American respondents in Los Angeles and Orange counties, 63.4%; and among Vietnamese American respondents in Los Angeles and Orange counties, 63.8%.

**Table 1:** Sociodemographic and Health Characteristics of Sample in Study on Racial/Ethnic Differences in Influenza (Flu) and Pneumococcal Vaccination Rates Among Adults Aged ≥50, by Location and Race/Ethnicity, 2009–2012^a^

Characteristic	% (95% Confidence Interval)							
	Non-Hispanic Black		Hispanic		Chinese American	Korean American	Filipino American	Vietnamese American
	New York City (n = 1,715)	Los Angeles and Orange Counties (n = 530)	New York City (n = 2,667)	Los Angeles and Orange Counties (n = 1,099)	New York City (n = 1,656)	New York City (n = 310)	Los Angeles and Orange Counties (n = 1,515)	Los Angeles and Orange Counties (n = 3,435)
**Flu vaccine (aged ≥50)**	53.3 (49.5–57.1)	40.5 (36.8–44.4)	61.0 (59.0–62.9)	49.4 (42.1–56.7)	67.6 (64.1–70.9)	60.5 (52.7–67.9)	66.2 (62.4–69.8)	68.0 (66.5–69.4)
**Pneumococcal vaccine (aged ≥65)**	62.0 (56.4–67.3)	65.6 (62.9–68.2)	60.0 (57.9–62.0)	62.7 (53.7–70.8)	51.7 (46.3–57.1)	49.1 (35.0–63.2)	63.4 (60.5–66.2)	63.8 (61.0–66.4)
**Age group**								
50–64	64.8 (61.3–68.1)	60.7 (53.3–67.7)	63.6 (55.4–71.2)	72.0 (66.2–77.1)	60.1 (57.2–63.0)	69.6 (65.6–73.3)	59.0 (54.2–63.5)	67.3 (64.7–69.7)
≥65	35.2 (31.9–38.7)	39.3 (32.3–46.7)	36.4 (28..8–44.7)	28.0 (22.9–33.8)	39.9 (37.0–42.8)	30.4 (26.8–34.4)	41.0 (36.5–45.8)	32.7 (30.3–35.3)
**Sex**								
Male	43.8 (39.2–48.5)	43.1 (36.9–49.6)	46.2 (40.0–52.5)	48.9 (45.7–52.1)	46.1 (42.8–49.4)	50.6 (48.7–52.5)	42.1 (40.2–44.2)	51.4 (48.5–54.2)
Female	56.2 (51.5–60.8)	56.9 (50.5–63.1)	53.8 (47.5–60.0)	51.1 (47.9–54.3)	53.9 (50.6–57.3)	49.4 (47.5–51.4)	57.9 (55.9–59.8)	48.6 (45.8–51.5)
**Nativity**								
Born in the United States	77.9 (69.7–84.4)	92.6 (86.6–96.0)	33.8 (27.8–40.3)	27.3 (23.4–31.6)	3.6 (2.6–4.9)	1.0 (0.3–2.8)	4.8 (3.7–6.1)	0.5 (0.3–0.7)
**Education**								
<High school diploma	24.0 (22.0–26.2)	6.3 (5.2–7.5)	49.9 (48.3–51.4)	49.8 (43.4–56.2)	50.9 (47.2–54.6)	12.6 (9.0–17.2)	3.6 (2.8–4.6)	25.3 (22.8–28.0)
High school diploma/some college	58.5 (56.1–60.9)	63.0 (57.2–68.5)	40.7 (38.8–42.5)	38.3 (33.8–43.0)	32.3 (30.6–34.0)	50.4 (44.8–56.0)	28.0 (26.3–29.9)	48.3 (45.9–50.7)
College graduate	17.5 (15.4–19.7)	30.7 (24.5–37.8)	9.5 (8.1–11.2)	11.9 (9.8–14.4)	16.8 (14.4–19.6)	37.0 (28.9–46.0)	68.3 (65.7–70.8)	26.4 (24.1–29.0)
**Self-reported health**								
Excellent/very good	27.1 (24.8–29.5)	36.0 (33.0–39.1)	16.9 (14.7–19.3)	23.6 (19.5–28.3)	17.8 (15.7–20.2)	32.2 (23.0–43.1)	32.1 (30.8–33.4)	21.7 (19.7–23.8)
Good	36.7 (34.4–39.2)	38.8 (35.0–42.6)	30.0 (28.0–32.1)	33.0 (27.8–38.6)	30.5 (24.3–37.4)	41.4 (33.3–50.0)	43.7 (42.9–44.5)	35.9 (33.8–38.0)
Fair/poor	36.2 (33.8–38.6)	25.3 (23.6–27.0)	53.1 (49.6–56.6)	43.4 (34.6–52.8)	51.7 (44.7–58.7)	26.4 (24.6–28.2)	24.3 (22.9–25.7)	42.5 (39.1–45.9)
**Health insurance**								
Yes	88.0 (86.5–89.3)	87.6 (84.1–90.4)	88.7 (87.2–90.1)	71.7 (67.3–75.8)	89.9 (87.4–91.9)	76.8 (74.7–78.8)	90.9 (89.6–92.0)	85.7 (83.9–87.2)
No	12.0 (10.7–13.5)	12.4 (9.6–15.9)	11.3 (9.9–12.8)	28.3 (24.2–32.7)	10.1 (8.1–12.6)	23.2 (21.2–25.3)	9.1 (8.0–10.4)	14.3 (12.8–16.1)
**Most recent checkup**								
Within the past year	83.9 (80.7–86.7)	79.4 (69.9–86.4)	83.7 (82.5–84.9)	71.2 (67.2–74.4)	84.3 (82.5–85.9)	65.6 (63.2–68.0)	80.8 (79.9–82.5)	77.9 (75.7–79.9)
More than a year/never/do not know	16.1 (13.3–19.3)	20.6 (13.6–30.1)	16.3 (15.1–17.5)	28.8 (25.6–32.3)	15.7 (14.1–17.5)	34.4 (32.0–36.9)	19.2 (17.5–21.1)	22.1 (20.2–24.3)
**Smoking status**								
Current	21.3 (19.4–23.3)	15.6 (12.7–18.8)	17.8 (16.0–19.8)	10.1 (8.6–11.9)	9.0 (7.4–10.8)	15.2 (12.1–19.0)	7.4 (5.5–9.3)	10.6 (9.6–11.6)
Former	30.6 (28.4–32.9)	33.5 (28.1–39.4)	26.7 (24.1–29.5)	26.1 (22.8–29.7)	15.5 (13.3–18.0)	25.9 (22.3–30.0)	19.9 (18.3–21.5)	18.5 (16.3–21.1)
Never	48.1 (45.0–51.3)	50.9 (47.1–54.7)	55.5 (53.0–58.0)	63.8 (59.1–68.2)	75.6 (73.1–77.9)	58.9 (58.3–59.5)	72.8 (70.3–75.1)	70.9 (67.6–74.0)
**Ever received diagnosis of stroke, angina, or coronary heart disease**								
Yes	15.7 (14.2–17.4)	14.6 (12.1–17.6)	18.2 (16.3–20.3)	11.4 (9.8–13.2)	11.6 (10.3–13.0)	10.8 (9.6–12.1)	11.6 (10.2–13.1)	10.6 (9.6–11.7)
No/do not know	84.3 (82.6–85.8)	85.4 (82.4–87.9)	81.8 (79.7–83.7)	88.6 (86.8–90.2)	88.4 (87.0–89.7)	89.2 (87.9–90.4)	88.4 (86.9–89.8)	89.4 (88.3–90.4)
**Needed a physician but was too costly**								
Yes	15.4 (12.8–18.3)	15.5 (13.6–17.5)	16.5 (15.0–18.0)	25.7 (21.4–30.7)	11.5 (10.8–12.2)	30.7 (26.7–35.0)	13.6 (11.9–15.5)	15.8 (14.3–17.5)
No	84.6 (81.7–87.2)	84.5 (82.5–86.4)	83.5 (82.0–85.0)	74.3 (69.4–78.6)	88.5 (87.8–89.2)	69.3 (65.0–73.4)	86.4 (84.5–88.1)	84.2 (82.5–85.7)
**Ever received a diabetes diagnosis**								
Yes	27.3 (25.1–29.7)	23.5 (21.6–25.4)	30.9 (27.8–34.3)	24.2 (21.7–26.9)	18.7 (16.9–20.6)	21.4 (18.1–25.0)	29.6 (28.3–31.0)	17.5 (16.7–18.3)
No	72.7 (70.3–74.9)	76.5 (74.6–78.4)	69.1 (65.7–72.2)	75.8 (73.1–78.3)	81.3 (79.4–83.1)	78.6 (75.0–81.9)	70.4 (69.0–71.7)	82.6 (81.7–83.3)

a The REACH US Risk Factor Survey was conducted annually from 2009 to 2012 to evaluate program activities in 28 communities (12).

Most non-Hispanic black respondents were born in the United States, whereas most Hispanic respondents and Asian American respondents were non-US born. Rates of health insurance were lowest among Hispanic respondents in Los Angeles and Orange counties (71.7%) and Korean American respondents in New York City (76.8%); rates of having a checkup within the past year were lowest among these same 2 groups and not being able to see a physician because it was too costly were highest. Non-Hispanic black respondents, Hispanic respondents, and Korean American respondents in New York City were most likely to be current smokers, whereas Filipino American respondents in Los Angeles and Orange counties were least likely to be current smokers. 

### Flu vaccination in New York City

In Model 1 of multivariable logistic regression predicting receipt of flu vaccination in the past year, Chinese American respondents were 1.7 times as likely (*P* < .001), Korean American respondents were 1.6 times as likely (*P* = .045), and Hispanic respondents were 1.3 times as likely (*P* = .01) as non-Hispanic black respondents to have received a flu vaccination (Table 2). Older age was significantly associated with receiving a flu vaccination (OR = 1.04, *P* < .001), whereas respondents with less than a high school diploma were 1.6 times as likely (*P* < .001) and those with a high school diploma or some college education were 1.2 times as likely (*P* = .02) as college graduates to have received a flu vaccination.

**Table 2:** Multivariable Logistic Regression Predicting Receipt of Influenza Vaccination Within Previous Year Among Samples of Adults Aged ≥50 in New York City and Los Angeles and Orange Counties, 2009–2012^a^

Characteristic	New York City		Los Angeles and Orange Counties	
	Model 1^b^	Model 2^c^	Model 1^b^	Model 2^c^
	OR (95% CI) [*P* Value]	OR (95% CI) [*P* Value]	OR (95% CI) [*P* Value]	OR (95% CI) [*P* Value]
**Race/ethnicity**				
Hispanic	1.3 (1.1–1.6) [.01]	1.2 (1.0–1.5) [.06]	2.3 (1.9–2.8) [<.001]	2.5 (1.9–3.4) [<.001]
Chinese	1.7 (1.3–2.2) [<.001]	1.8 (1.3–2.5) [<.001]	—d	—d
Korean	1.6 (1.0–2.5) [.045]	2.2 (1.4–3.7) [.003]	—d	—d
Filipino	—d	—d	4.2 (2.9–6.0) [<.001]	4.0 (2.5–6.5) [<.001]
Vietnamese	—d	—d	5.5 (4.0–7.6) [<.001]	5.6 (3.6–8.8) [<.001]
Non-Hispanic Black	1 [Reference]	1 [Reference]	1 [Reference]	1 [Reference]
**Age, continuous**	1.04 (1.03–1.05) [<.001]	1.03 (1.02–1.04) [<.001]	1.08 (1.07–1.09) [<.001]	1.06 (1.05–1.07) [<.001]
**Sex**				
Female	1.1 (1.0–1.2) [.29]	0.9 (0.8–1.0) [.11]	1.2 (1.0–1.4) [.04]	1.2 (1.0–1.4) [.053]
Male	1 [Reference]	1 [Reference]	1 [Reference]	1 [Reference]
**Nativity**				
US born	1.0 (0.9–1.2) [.71]	1.0 (0.9–1.2) [.87]	1.3 (1.1–1.6) [.007]	1.2 (1.1–1.5) [.01]
Non-US born	1 [Reference]	1 [Reference]	1 [Reference]	1 [Reference]
**Education**				
<High school	1.6 (1.3–1.9) [<.001]	1.4 (1.2–1.7) [.001]	0.9 (0.7–1.1) [.42]	0.9 (0.8–1.1) [.45]
High school/some college	1.2 (1.0–1.4) [.02]	1.1 (0.9–1.4) [.20]	1.0 (0.9–1.2) [.82]	1.0 (0.9–1.2) [.75]
College graduate	1 [Reference]	1 [Reference]	1 [Reference]	1 [Reference]
**Insurance coverage**				
Yes	—e	1.8 (1.4–2.2) [<.001]	—e	2.1 (1.9–2.4) [<.001]
No		1 [Reference]		1 [Reference]
**Most recent checkup**				
Within the past year	—e	2.5 (2.1–2.9) [<.001]	—e	2.0 (1.8–2.3) [<.001]
More than a year/never/don’t know		1 [Reference]		1 [Reference]
**Self-reported health**				
Good	—e	1.2 (1.0–1.3) [.02]	—e	1.2 (1.1–1.4) [.003]
Fair/poor		1.4 (1.1–1.7) [.02]		1.4 (1.3–1.6) [<.001]
Excellent/very good		1 [Reference]		1 [Reference]
**Ever received diagnosis of stroke, angina, or coronary heart disease**				
Yes	—e	1.2 (1.0–1.5) [.054]	—e	1.4 (1.0–1.9) [.03]
No/don’t know		1 [Reference]		1 [Reference]
**Ever received a diabetes diagnosis**				
Yes	—e	2.0 (1.8–2.3) [<.001]	—e	1.6 (1.4–1.8) [<.001]
No		1 [Reference]		1 [Reference]

a The REACH US Risk Factor Survey was conducted annually from 2009 to 2012 to evaluate program activities in 28 communities (12).

b Model 1 accounted for sociodemographic variables.

c Model 2 (the full model) accounted for sociodemographic and health-related variables.

d Asian subgroups with <100 respondents were not included in analysis.

e Model 1 did not account for health-related variables.

In Model 2, both Chinese American respondents (OR = 1.8, *P* < .001) and Korean American respondents (OR = 2.2, *P* = .003) were more likely than non-Hispanic black respondents to have received a flu vaccination; Hispanic ethnicity was no longer significant. Older age and having less than a high school diploma remained significant. Additional factors associated with receiving a flu vaccination were having health insurance (OR = 1.8, *P* < .001), having had a checkup within the past year (OR = 2.5, *P* < .001), self-reporting health as good (OR = 1.2, *P* = .02) or fair/poor (OR = 1.4, *P* = .02), and self-reported diabetes (OR = 2.0, *P* < .001).

### Flu vaccination in Los Angeles and Orange counties

In Model 1, Vietnamese American respondents were 5.5 times as likely (*P* < .001), Filipino American respondents were 4.2 times as likely (*P* < .001), and Hispanic respondents were 2.3 times as likely (*P* < .001) as non-Hispanic black respondents to have received a flu vaccination. Similar to New York City findings, older age was significantly associated with receiving a flu vaccination (OR = 1.08, *P* < .001). In addition, women were 1.2 times as likely as men to have received a flu vaccination (*P* = .04), and US-born respondents were 1.3 times as likely as non–US-born respondents to have received a flu vaccination (*P* = .007).

In Model 2, Vietnamese American respondents were 5.6 times as likely (*P* < .001), Filipino American respondents were 4.0 times as likely (*P* < .001), and Hispanic respondents were 2.5 times as likely (*P* < .001) as non-Hispanic black respondents to have received a flu vaccination. Older age and US nativity remained significant. Additional factors associated with receiving a flu vaccination were having health insurance (OR = 2.1, *P* < .001); having had a checkup within the past year (OR = 2.0, *P* < .001); self-reporting health as good (OR = 1.2, *P* = .003) or fair/poor (OR = 1.4, *P* < .001); a diagnosis of stroke, angina, or coronary heart disease (OR = 1.4, *P* = .03); and self-reported diabetes (OR = 1.6, *P* < .001).

### Pneumococcal vaccination in New York City

Racial/ethnic minority group was not significantly associated with pneumococcal vaccination in either multivariable logistic regression predicting pneumococcal vaccination in lifetime (Table 3). In Model 1, only older age was associated with ever receiving the pneumococcal vaccination (OR = 1.01, *P* = .02). 

**Table 3:** Multivariable Logistic Regression Predicting Receipt of Pneumococcal Vaccination In Lifetime Among Samples of Adults Aged ≥65 in New York City and Los Angeles and Orange Counties, 2009–2012^a^

Characteristic	New York City		Los Angeles and Orange Counties	
	Model 1^b^	Model 2^b^	Model 1^b^	Model 2^c^
	OR (95% CI) [*P* Value]	OR (95% CI) [*P* Value]	OR (95% CI) [*P* Value]	OR (95% CI) [*P* Value]
**Race/ethnicity**				
Hispanic	1.1 (0.8–1.3) [.67]	1.0 (0.8–1.4) [.88]	1.2 (0.8–1.9) [.40]	1.3 (0.8–2.1) [.37]
Chinese	0.7 (0.5–1.0) [.08]	0.7 (0.5–1.1) [.11]	—d	—d
Korean	0.8 (0.5–1.4) [.39]	0.8 (0.4–1.5) [.45]	—d	—d
Filipino	—d	—d	1.1 (0.8–1.5) [.67]	1.1 (0.7–1.7) [.62]
Vietnamese	—d	—d	1.2 (0.9–1.6) [.19]	1.2 (0.8–1.9) [.30]
Non-Hispanic Black	1 [Reference]	1 [Reference]	1 [Reference]	1 [Reference]
**Age (continuous)**	1.01 (1.00–1.03) [.02]	1.01 (1.00–1.03) [.09]	1.04 (1.02–1.07) [<.001]	1.04 (1.02–1.07) [<.001]
**Sex**				
Female	1.1 (1.0–1.3) [.11]	1.1 (0.9–1.3) [.38]	1.1 (1.0–1.2) [.06]	1.1 (1.0–1.2) [.20]
Male	1 [Reference]	1 [Reference]	1 [Reference]	1 [Reference]
**Nativity**				
Non-US born	1.2 (1.0–1.5) [.05]	1.3 (1.0–1.6) [.046]	1.1 (0.8–1.4) [.66]	1.1 (0.7–1.6) [.81]
US born	1 [Reference]	1 [Reference]	1 [Reference]	1 [Reference]
**Education**				
< High school	0.9 (0.8–1.2) [.50]	0.9 (0.7–1.1) [.22]	0.6 (0.5–0.7) [<.001]	0.6 (0.4–0.7) [<.001]
High school/Some college	0.9 (0.7–1.2) [.60]	0.9 (0.7–1.2) [.39]	0.9 (0.8–1.1) [.22]	0.9 (0.8–1.1) [.24]
College graduate	1 [Reference]	1 [Reference]	1 [Reference]	1 [Reference]
**Health care coverage**				
Yes	—e	1.5 (0.9–2.3) [.08]	—e	1.8 (1.2–2.8) [.006]
No		1 [Reference]		1 [Reference]
**Most recent checkup**				
Within the past year	—e	1.7 (1.3–2.1) [<.001]	—e	1.7 (1.4–2.1) [<.001]
More than a year/never/don’t know		1 [Reference]		1 [Reference]
**Self-reported health**				
Good	—e	1.3 (0.9–1.9) [.12]	—e	1.2 (1.0–1.4) [.03]
Fair/poor		1.4 (1.1–1.8) [.009]		1.2 (0.9–1.5) [.21]
Excellent/very good		1 [Reference]		1 [Reference]
**Ever received diagnosis of stroke, angina, or coronary heart disease**				
Yes	—e	1.4 (1.1–1.8) [.008]	—e	1.0 (0.7–1.5) [.86]
No/don’t know		1 [Reference]		1 [Reference]
**Ever received a diabetes diagnosis**				
Yes	—e	1.6 (1.4–1.8) [<.001]	—e	1.2 (1.0–1.5) [.02]
No		1 [Reference]		1 [Reference]

a The REACH US Risk Factor Survey was conducted annually from 2009 to 2012 to evaluate program activities in 28 communities (12).

b Model 1 accounted for sociodemographic variables.

c Model 2 (the full model) accounted for sociodemographic and health-related variables.

d Asian subgroups with <100 respondents were not included in analysis.

e Model 1 did not account for health-related variables.

In Model 2, age was no longer significant. Additional factors associated with having ever received a pneumococcal vaccination were being non-US born (OR = 1.3, *P* = .046), having had a checkup within the past year (OR = 1.7, *P* < .001), self-reporting health as fair/poor (OR = 1.4, *P* = .009), self-reported stroke, angina, or coronary heart disease (OR = 1.4, *P* = .008), and self-reported diabetes (OR = 1.6, *P* < .001).

### Pneumococcal vaccination in Los Angeles and Orange counties

Racial/ethnic subgroup was not significantly associated with pneumococcal vaccination in either model. In Model 1, older age was significantly associated with having ever received a pneumococcal vaccination (OR = 1.04, *P* < .001). Respondents with less than a high school diploma were less likely than respondents who were college graduates to have ever received a pneumococcal vaccination (OR = 0.6, *P* < .001).

In Model 2, age and education remained significant. Additional factors associated with having ever received the pneumococcal vaccine were having health insurance (OR = 1.8, *P* = .006), having had a checkup within the past year (OR = 1.7, *P* < .001), self-reporting health as good (OR = 1.2, *P* = .03), and self-reported diabetes (OR = 1.2, *P* = .02).

## Discussion

Our study results indicate that among non-Hispanic black, Hispanic, and Asian respondents to the REACH US Risk Factor Survey 2009–2012, non-Hispanic black respondents were the least likely to receive a flu vaccination in New York City and Los Angeles and Orange counties; however, we found no significant association between pneumococcal vaccination rate and race/ethnicity in New York City or Los Angeles and Orange counties. Our study also identified 4 variables that are significantly associated with positive outcomes for both flu and pneumococcal vaccination in both locations: older age (not significant in pneumococcal vaccine Model 2, New York City), having had a checkup in the past year, self-reported fair/poor health, and self-reported diabetes. All these indicators were associated with flu and pneumococcal vaccination in previous studies (4,5,11,21). One possible explanation for the association between self-reported diabetes and higher rates of flu and pneumococcal vaccination is that people with diabetes, who may have a weaker immune response to infections, are at higher risk of developing serious complications caused by flu and pneumonia infections (22). Primary care providers may recommend these vaccines to prevent further illness.

In addition to identifying shared variables that are associated with positive vaccination outcomes, our findings also distinguished variables that are uniquely associated with each vaccination type in each geographic location. For example, lower educational attainment was significantly associated with flu vaccine receipt in New York City, and lower educational attainment was inversely associated with pneumococcal vaccine receipt in Los Angeles and Orange counties. Additionally, US birthplace was significantly associated with a positive flu vaccination outcome in Los Angeles and Orange counties, whereas a non-US birthplace was significantly associated with pneumococcal vaccination outcome in New York City. Lastly, health insurance coverage was significantly associated with a positive flu and pneumococcal vaccination outcome in Los Angeles and Orange counties and with a positive flu vaccination outcome in New York City. Insurance, particularly private insurance, is associated with flu and pneumococcal vaccination (4,5,21).

Similar to previous studies that showed racial/ethnic disparities in flu and pneumococcal vaccination rates among non-Hispanic black adults, compared with non-Hispanic white adults (4,5,21,23), our study findings also showed racial/ethnic differences in flu vaccination rates. Non-Hispanic black adults had the lowest flu vaccination rate when compared with Hispanic adults and Asian American subgroups. Unlike previous studies, our findings did not indicate a significant association between race/ethnicity and pneumococcal vaccination. However, our study did not include non-Hispanic white adults, who may report higher vaccination rates. Additionally, we focused only on adults aged 50 or older, whereas previous studies described overall flu vaccination rates among all adults (aged ≥18) or adults aged 65 or older.

Similar to a previous study comparing vaccination rates among Vietnamese American, Asian American, and non-Hispanic white adults aged 18 or older in Santa Clara County, California (11), our study also identified older age, having had a recent checkup, and self-reported diabetes to be significantly associated with a positive vaccination outcome. Conversely, the association between self-reported fair/poor health and vaccination outcome is unique to our study findings for Asian Americans, although it was significant in a study of non-Hispanic black and non-Hispanic white Medicare beneficiaries (4).

Our study has several limitations. First, we excluded from analysis Asian American subgroups with a sample size of fewer than 100; because Asian American subgroups differed by location, we were unable to compare data from the same subgroups in both locations. Larger sample sizes for Asian American subgroups are needed in future research. Second, our study used self-reported data; therefore, our findings may be subject to recall bias. Finally, we were not able to assess differences in vaccination rates among unique subgroups of non-Hispanic black and Hispanic respondents. Future research should expand options on surveys, allowing respondents to pinpoint their race/ethnicity, so that disparities can be further investigated across subgroups.

The strengths of this study include the comparison of multiple racial/ethnic groups, the disaggregation of data on Asian Americans, and the comparison of vaccination rates between 2 locations. Although previous studies established that disparities in vaccination rates exist between non-Hispanic white adults and non-Hispanic black adults, and between non-Hispanic white adults and Asian American adults, no previous studies compared non-Hispanic black adults with Asian American adults, Hispanic adults with Asian American adults, and most importantly, various Asian American subgroups. Our study disaggregated Asian American respondents into 4 subgroups, allowing for comparison across subgroups, as well as with Hispanic and non-Hispanic black respondents. Additionally, our data compared vaccination rates between New York City and Los Angeles and Orange counties, which may provide insights to differences and similarities in vaccination barriers.

Our study identified 4 variables that are strongly associated with positive vaccination outcomes; these factors may help guide implementation of health interventions to effectively reach target populations and communities. Treatment teams may consider age, most recent checkup, self-reported health, and diabetes diagnosis when developing primary interventions to maximize protection against vaccine-preventable diseases. CDC suggests increasing coverage by expanding access through nontraditional settings such as pharmacies and by improving the use of evidence-based practices such as reminder/recall notifications (24). These recommendations can also be modified to accommodate the needs of racial/ethnic minority older adults. For example, if these adults are unable to visit a traditional clinical setting, then immunizations can be offered at local community centers or through a home visit by a health care provider. Reminder/recall notifications are methods to identify and notify patients that immunizations are due or behind (25). Reminder messages can be recorded in multiple languages and designed to educate recipients on the importance of immunization regardless of health status. Finally, involving family members and caregivers who may have more advanced skills in the use of technological devices may help expand reminder/recall benefits among older adults.

Our study findings demonstrate that vaccination rates among older racial/ethnic populations living in New York City and Los Angeles and Orange counties are suboptimal and that disparities exist among these groups. More granular data on racial/ethnic subgroups are needed. Routine monitoring and reporting of disaggregated vaccine coverage by race, ethnicity, and sociodemographic factors are needed to identify cultural barriers that are unique to each race/ethnicity and factors associated with vaccination outcomes that may not be measureable when coverage data are aggregated (5).
